# The Expression of GHS-R in Primary Neurons Is Dependent upon Maturation Stage and Regional Localization

**DOI:** 10.1371/journal.pone.0064183

**Published:** 2013-06-05

**Authors:** Donatella Lattuada, Katia Crotta, Noemi Tonna, Claudia Casnici, Roberta Benfante, Diego Fornasari, Fabio Bianco, Renato Longhi, Ornella Marelli

**Affiliations:** 1 Department of Medical Biotechnologies and Translational Medicine, University of Milan, Milan, Italy; 2 NeuroZone srl, Milano, Italy; 3 CNR – Institute of Neuroscience, Milan, Italy; 4 CNR, Istituto di Chimica del Riconoscimento Molecolare, Milan, Italy; University of Sydney, Australia

## Abstract

Ghrelin is a hormone with a crucial role in the regulation of appetite, regulation of inflammation, glucose metabolism and cell proliferation. In the brain ghrelin neurons are located in the cortex (sensorimotor area, cingular gyrus), and the fibres of ghrelin neurons in hypothalamus project directly to the dorsal vagal complex (DVC). Ghrelin binds the growth hormone secretagogue receptor (GHS-R) a G-protein-coupled receptor with a widespread tissue distribution, indeed these receptors are localized both in nonnervous, organs/tissues (i.e. adipose tissue, myocardium, adrenals, gonads, lung, liver, arteries, stomach, pancreas, thyroid, and kidney) as well as in central nervous system (CNS) and higher levels of expression in the pituitary gland and the hypothalamus and lower levels of expression in other organs, including brain. A GHS-R specific monoclonal antibody has been developed and characterized and through it we demonstrate that GHS-R is expressed in primary neurons and that its expression is dependent upon their developmental stage and shows differences according to the brain region involved, with a more pronounced expression in hippocampal rather than cortical neurons. A characterization of GHS-R within the central nervous system is of extreme importance in order to gain insights on its role in the modulation of neurodegenerative events such as Alzheimer’s disease.

## Introduction

Ghrelin is a multifunctional 28-amino acid (aa) hormone produced in a wide variety of tissues, including the brain, where it can act as a paracrine/autocrine factor [1].

Ghrelin was originally identified based on its ability to stimulate GH release. However, subsequent studies demonstrated that the ghrelin system is involved in a number of divergent functions such as regulation of food intake, body weight gain, insulin release and β-cell survival, adiposity, and the control of energy homeostasis [2], [3], as well as, it participates in many other physiological processes such as circulation, cell proliferation, differentiation and apoptosis [4], [5]. Likewise, the ghrelin system has also been shown to be involved in inflammation [6], [7] and modulation of neuronal functions [8]–[10].

In the brain ghrelin is present in the hypothalamic arcuate nucleus (ARC), where it is especially abundant in the ventral part, an important region in the control of appetite [11]. Ghrelin neurons are located also in the cortex (sensorimotor area, cingular gyrus), and the fibres of ghrelin neurons in hypothalamus project directly to the dorsal vagal complex (DVC) [12].

Ghrelin binds the growth hormone secretagogue receptor (GHS-R), a G-protein-coupled receptor, localized both in nonnervous, organs/tissues (i.e. adipose tissue, myocardium, adrenals, gonads, lung, liver, arteries, stomach, pancreas, thyroid, and kidney) as well as in central nervous system (CNS). It has been demonstrated that GHS-R shows different levels of expression in different tissues [13]–[19].

GHS-R is prominently expressed in different regions of the brain. Indeed, GHS-R mRNA has been reported in the ARC and ventromedial nuclei (VMN) and in CA2 and CA3 regions of the hippocampus, in the substantia nigra, the ventral tegmental area, the dentate gyrus of the hippocampal formation, and the dorsal and median raphe nuclei [15], [20].

Ghrelin has been shown to regulate brain functions such as modulation of cognitive processes, not only in the hypothalamus but also in other brain areas with stimulatory effect on memory retention through promotion of synaptic plasticity [8], and generation of long-term potentiation [21], [22], [23]. Interestingly, these ghrelin-induced synaptic changes were closely paralleled by enhanced hippocampus-dependent spatial learning and memory [10].

Studies concerning the neuroprotective role of ghrelin were carried out in hypothalamus, in a model of rat injury, where it was able to significantly increase the number of surviving neurons and reduce the number of apoptotic neurons in CA1 area of the hippocampus [24].

Subsequently, in vitro studies on primary hypothalamic neurons exposed to oxygen–glucose deprivation protocol (OGD) further supported a neuroprotective role of ghrelin. Specifically, ghrelin exerted their actions by inhibiting generation of reactive oxygen species and stabilizing mitochondrial transmembrane potential. In addition, ghrelin-treated neurons showed an increased Bcl-2/Bax ratio, a reduced cytochrome c release, and reduced caspase-3 activation [25]. Moreover, similar to hypothalamic neurons, ghrelin exerts its neuroprotection in cortical neurons by inhibiting pro-apoptotic molecules associated with mitochondrial pathways and by activating endogenous protective molecules [26].

Overall, evidence so far collected suggests a crucial role for ghrelin in the modulation of several phenomena associated with aging processes, such as development of reactive oxygen species, memory loss and onset of neuroinflammatory scenarios. Therefore, aim of this work is study the modulation of GHS-R levels of expression in neurons at different stages of development and obtained of different brain regions. In order to do that we produced and characterized a monoclonal antibody specific for the N-terminal region of GHSR.

## Materials and Methods

### Sequence Analysis

Monoclonal antibody sequence was analysed by FASTA from European Bioinformatics Institute to exclude the existence of human membrane proteins with significant sequence homology.

#### GHSR N-Terminal human sequence

The ghrelin receptor (GHS-R) amino acid sequence. Monoclonal antibody sequence (here undescored) is a portion of N-terminal sequence of GHS-R and is a shared sequence between the two GHSR isoforms 1a and 1b:

**MWNATPSEEPGFNLTLADLDWDASPG**NDSLGDELLQLFPAPLLAGVTATC VALFVVGIAGNLLTMLVVSR FRELRTTTNL YLSSMAFSDLIFLCMPLDL VRLWQYRPWN FGDLLCKLFQ FVSESCTYAT VLTITALSVE RYFAICFPLR AKVVVTKGRVKLVIFVIWAVAFCSAGPIFVLVGVEHENGTDPWDTNECRPTEFAVRSGLLTVMVWVSSIFFFLPVFCLTVLYSLIGRKLWRRRRGDAVVGASLRDQNHKQTVKMLAVVVFAFILCWLPFHVGRYLFSKSFEPGSLEIAQISQYCNLVSFVLFYLSAAINPILYNIMSKKYRVAVFRLLGFEPFSQRKLSTLKDESSRAWT.

### Primary Cultures of Hippocampal/cortical Neuron and Astrocytes

Primary neuronal cultures were prepared from the brains of 20 day-old rat embryos (Charles River) as previously described [27] with minor modifications. Briefly, the hippocampi or cortices were isolated from total brain, incubated with trypsin at 37°C, and then dissociated in order to obtain separated cells, which were then plated at ranging density from 10,000 to 20,000 cells/cm^2^ on glass cover slips previously coated with poly-lysine (Sigma Aldrich) and grown in Neurobasal medium (Gibco Invitrogen) supplemented with B27 (Gibco Invitrogen), 0.5 mM glutamine and 12.5 µM glutamate. All experiments were performed in accordance with the guidelines established in Fondazione Filarete Campus Principles of Laboratory Animal Care (directive 86/609/EEC). The protocol was approved by Italian Health Ministry (art 12 D.L.vo n. 116/92 – Decreto n. 23/2010-A del 01/02/2010. All efforts were made to minimize suffering. Animals were anesthetized and sacrificed as indicated by European guidelines CPMP/ICH/302/9.

### Immunization Protocol

Six male CD_2_F_1_ mice (Charles River), 7–12 weeks old, were immunized subcutaneously four times at 2-week intervals with 100 µg of peptide/mouse. Peptide conjugated with Keyhole Limpet Hemocyanin (KLH) was emulsified in the same volume of Freund’s Adjuvant (Sigma). For priming, the emulsion was prepared with Complete Freund’s Adjuvant. Mice were housed in appropriate animal care facilities and handled according to international guidelines for experiments with animals.

### Generation of GHS-R Hybridomas

After four booster injections test bleeds were assayed for positive reactions to peptide GHS-R by indirect enzyme-linked immunoabsorbant assay (ELISA). Two weeks later followed an i.v. boost of 75 µg of peptides in normal saline solution and three days post boost spleen cells from immunized mice were fused to P3×63Ag8.653 mouse myeloma cells (Biological Bank, Istituto Nazionale per la Ricerca sul Cancro IST, Genova, Italy) in the presence of a 50% solution (wt/ml) of polyethylene glycol (Molecular Weight 3350, Sigma) to produce hybridoma cells according to standard procedures [28]. Cells were plated in 96-well plates (Corning-Costar Corp) and cultured at 37°C in a humidified atmosphere in the presence of 5% CO_2_ and 95% air in RPMI 1640 medium supplemented with 20% Fetalclone I (Hyclone,Thermo Scientific), L-glutamine (Euroclone), penicillin, streptomycin (Sigma), hypoxanthine-aminopterin-thymidine (Sigma) 100 µM, 0.4 µM and 16 µM respectively to select hybrid cells. Supernatants from the growing hybridomas were screened with ELISA. Positive wells were sub-cloned by limiting dilution and tested by indirect ELISA. Selected hybridoma lines were later grown and isotypes of the Mab were determined using the Rapid Isotyping kit (Thermo Scientific) according to the manufacturer’s instructions. Concentrated MAb supernatants were purified using Protein L Sepharose Fast Flow (Sigma).

### Antibody Purification

Cell culture supernatant was centrifuged at 1270 g for 30 min. Saturated ammonium sulphate solution was added to the supernatant to bring the final concentration to 50% saturation and incubated at 4°C overnight. The solution was centrifuged for 30 min at 1270g and the pellet resuspended in 10% of the starting volume of phosphate-buffered saline. The antibody solution was dialysed versus two changes of PB overnight. 4 ml of immobilized Protein L agarose Flow (Sigma) were packed into a suitable column under gravity flow. The column was equilibrated in 0.1M sodium phosphate buffer pH 7.2, containing 0.15 M NaCl (Binding Buffer). The dialysed solution was diluted 1∶1 with Binding Buffer and loaded onto the column. The column was then washed with 15 ml of Binding Buffer and then stripped by washing with 0.1 M glycine, pH 2.7. Eluated fractions containing antibody were collected, immediately adjusted to pH 7.5 by adding TRIS 1 M pH 9 and tested by means of ELISA.

### ELISA

ELISA 96-well plates (Immobilizer – Nunc Streptavidin) were washed three times with PBS+0.05% Tween-20 (Sigma-Aldrich) (PBST) and coated with 1 µg/w of peptide GHS-R diluted in PBST at room temperature (RT) for 1 h with agitation. Plates were washed four times with PBST and supernatants (50 µl) from hybridoma cultures, immune serum, or purified antibody diluted in PBST were added and incubated at RT for 2 h with agitation. Plates were washed four times with PBST before incubation with secondary goat anti-mouse Ig antibody conjugated to alkaline phosphatase (BD Pharmingen) at RT for 1 h. After further washes with PBST the plates were incubated with p-nitrophenyl phosphate (Sigma) diluted to 1 mg/mL in diethanolamine substrate buffer (Thermo Scientific) at RT for 60 min with agitation. The optical density at 405 nm was detected on a Victor3 plate reader (Perkin Elmer).

### Total RNA Extraction and Reverse Transcription

Total RNA was extracted from a maximum of 7×10^5^ hippocampal and cortical primary neurons isolated from two independent rats and kept in culture for 4, 9, 16 and 21 days, using the RNeasy™ Mini Kit and accompanying QIAshredder™ (Qiagen) according to the manufacturer’s instructions. Subsequently, 0.2 µg was reverse transcribed using the SuperScript™ III First-Strand Synthesis System for RT-PCR (Invitrogen) in accordance with the manufacturer’s instructions and analysed for the expression of the Grelin Receptor using real-time PCR.

### Quantitative Real-time PCR

Gene expression was quantitatively analysed using the ABI Prism™ 7000 Sequence Detection System (Applied Biosystems) and SDS software version 1.2.3. The target sequences were amplified from 10 ng of cDNA in the presence of TaqMan® Gene Expression Master Mix. The TaqMan® primer and probe assays used were rat *GHS-R* (ID #Rn00821417_m1*), and the endogenous controls *GADPH* (ID #Rn01775763_g1*). The 2^−ΔCT^ method was used to calculate the results, thus allowing the normalisation of each sample to the endogenous control.

### Immunocytochemical Staining

Primary neurons at different stages of development (4, 9, 16, 21 days “in vitro”) were fixed in 4% paraformaldehyde and 4% sucrose at room temperature (RT), for 10 min. Primary and secondary antibodies were applied in GDB buffer (30 mM phosphate buffer, pH 7.4, containing 0.2% gelatin, 0.5% Triton X-100, and 0.8 M NaCl) for 2 hr at RT. The confocal images were acquired with a Leica SPE confocal microscope, using a Nikon (Tokyo, Japan) 40× objective with a sequential-acquisition setting at a resolution of 1024×1024 pixels. Each image was a *z*-series projection taken at 0.8 µm deep intervals.

The following antibodies were used: Polyclonal abs against VGlut-1 (1∶1000 dilution) was from Synaptic System (Gottingen), Secondary antibody Alexa 546 was from Life Technologies.

### Immunoprecipitation and Western Blot Analysis

For immunoprecipitation experiments, cells were lysed with lysis buffer containing 20 mM Na_2_HPO_4_ (pH 7.2), 150 mM NaCl, 2 mM EGTA, 25 mM NaF, 1 mM Na_3_VO_4,_ protease inhibition cocktail (Sigma), 1% Triton X-100, 0.5% saponine (Sigma). Lysates were clarified by centrifugation at 15900 g at 4°C for 30 min. The BCA protein assay kit (Thermo Scientific) was used to determine protein concentrations.

Clarified lysates were incubated with Mab GHS-R (8 µg) or polyclonal antibody at 4°C overnight, then bound to protein L agarose (Sigma) at 4°C for 2 h. The agarose was washed three times with lysis buffer and proteins were released from the agarose by boiling in NuPage LDS Sample buffer (Invitrogen) for 5 min. The proteins were then subjected to SDS-PAGE and transferred to 0.45 µm nitrocellulose membranes (Invitrogen) at 30 Volts for 110 minutes at 4°C. After washing three times with PBS+0.1% Tween and twice with distilled water, the blots were incubated at 4°C overnight with purified commercial polyclonal (Santa Cruz) or monoclonal antibody specific for GHS-R diluted in blocking solution (PBS+0.1% Tween+5% Skim milk). The membranes were washed three times with PBS+0.1% Tween and incubated with goat anti-rabbit IgG conjugated to horseradish peroxidase (BIO-RAD) at room temperature for 45 min. Following three washes with PBS+0.1% Tween and two with distilled water, the antibody-reactive bands were visualized using chemiluminescence with ECL and exposure to film (Hyperfilm ECL; Amersham).

### Cytofluorimetric Analysis

For cell surface staining, the cells were incubated with FCS at room temperature for 30 min. 0.5×10^6^ cells were then incubated on ice for 30 min with purified Mab (6 µg) in FACS buffer (PBS containing 5% FCS and 0.1% sodium azide, 100 µL/sample). After three washes with FACS buffer, cells were stained with phycoerythrin-conjugated anti-mouse Ig (Dako) diluted 1∶10 in FACS buffer. Incubation was carried out in the dark at 4°C for 30 min and was followed by three washes with FACS buffer. Cells were analyzed by FACSCalibur (Becton Dickinson). Analysis was carried out using a CellQuest software package (Becton Dickinson). For all samples, 20,000 events were acquired in the R1 region gate, which was defined based on forward and side light scatter properties to exclude debris.

### siRNA Analysis

In order to carry out GHS-R selective silencing RNA experiments, we used commercially available probes for GHS-R siRNA (Ambion selected pre-designed siRNA, Life Technologies). Transfection was carried out using Lipofectamine (Life Technologies) following manufacturer’s protocol. Cells at the time of transfection were cultured on 16 mm diameter coverglass at 60% confluency. The following volumes of reagents were taken into consideration: total transfection volume 1 ml, siRNA 15 pMol, DNA e GFP 0.4 µg, transfection agent 3 µl. Cells were exposed to transfection agent/siRNA/DNA complex for 1 hour. Following incubation, cells were washed in Dulbecco’s medium without phenol red and subsequently cultured in standard conditions. 48 hrs later, cells were fixed in 4% paraformaldehyde and 4% sucrose at room temperature (RT), for 10 min. Finally, immunocytochemical staining protocol was carried out, and cells were stained for GHS-R. In order to verify silencing efficacy, real time quantitative PCR was carried out on transfected cells lysate as previously described.

## Results

**Figure 1:**
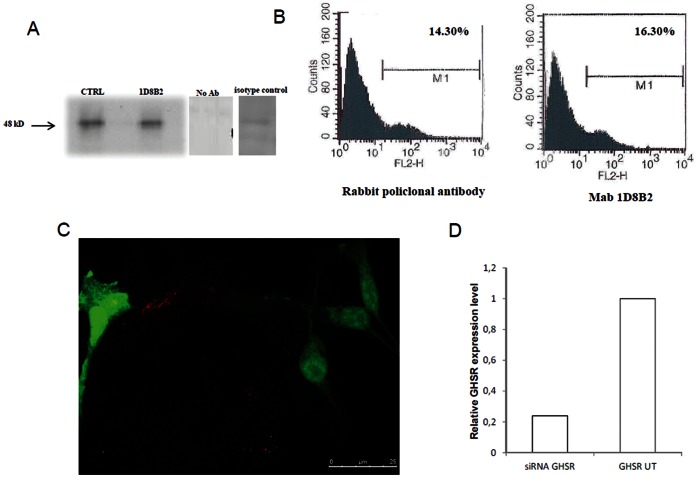
Production and characterization of Mab anti-GHS-R. A. Immunoprecipitation of 22RV1 cell lysates with Mab 1D8B2 and commercial polyclonal antibody (CTRL). Lysates from cells were immunoprecipitated with Mab (8 µg) or polyclonal antibody, resolved and transferred to nitrocellulose membranes. The western blot analysis were performed with the polyclonal antibody when the monoclonal was used for immunoprecipitation and the other way around, both immunoprecipitation analysis clearly showed a 48 kD band corresponding to the predicted size of GHS-R. Sizes (kD) of molecular mass markers are indicated on the left. These experiments were performed independently at least twice with similar results. B. Binding analysis of Mab 1D8B2 to 22RV1 cells by flow cytometry. Purified monoclonal antibody (6 µg) were analyzed with a flow cytofluorimetric analysis to assess the binding of antibodies to the GHSR on the surface of the cells. The control consisted of an anti-GHSR purified polyclonal antibody. Experiments were performed four times with reproducible results C. Representative image of 22VR1 cells cotransfected with GHS-R siRNA and eGFP. eGFP (green) expressing cells do not show positivity for GHSR (red). D. Relative mRNA expression of GHS-R in transfected 22VR1 normalized on GAPDH. GHS-R expression is significantly lower in transfected cells(siRNA GHSR) than in non-transfected cells (GHSR UT).

**Figure 2:**
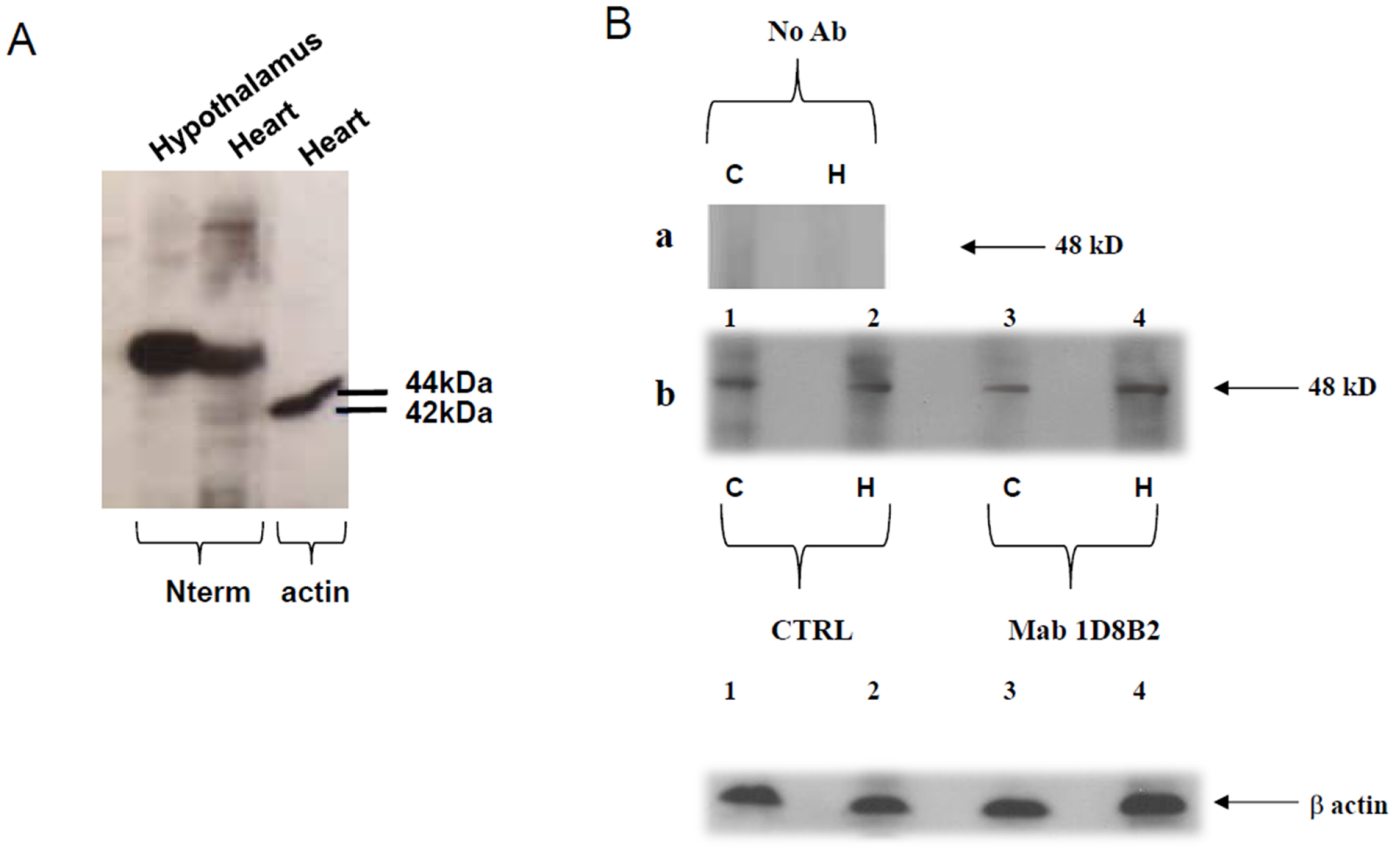
Evaluation of anti-GHS-R Mab specificity. A. Western blot of analysis of a selected brain region (hypothalamus) and a non-brain GHS-R bearing tissue (heart) showing a single band of the predicted molecular weight for GHSR at 48 kDa B. Immunoprecipitation of rat cortical (C) and hippocampal (H) primary neurons lysates with Mab 1D8B2 and commercial polyclonal antibody. Lysates from rat hippocampal and cortical neurons after nine days in vitro were immunoprecipitated with Mab (8 µg) or polyclonal antibody (CTRL), resolved and transferred to nitrocellulose membranes. The western blot analysis were performed with the polyclonal antibody when the monoclonal was used for immunoprecipitation and the other way around, both immunoprecipitation analysis clearly showed a 48 kD band corresponding to the predicted size of GHS-R. β-actin was used as a loading control. Sizes (kD) of molecular mass markers are indicated on the right.

Mab anti-GHS-R was produced immunizing male CD_2_F_1_ mice, with peptide sequence as described in the Material and Methods section. The amino acid sequence recognized by monoclonal antibodies anti-GHS-R is a portion of N-terminal sequence of GHS-R. Furthermore the antigen peptide used to immunize mice was screened by FASTA from European Bioinformatics Institute, with the aim of excluding the existence of other human or rat membrane proteins with significant homology. All mice developed an antibody titre of approximately >1∶2000, as tested by binding to the unconjugated peptides by ELISA. The mouse developing the highest antibody titre was selected for fusion with P3X63Ag8.653 mouse myeloma cells. All clones were screened by ELISA and only anti-GHS-R producing hybridomas whose supernatants responded with an OD value >0.4 versus negative control were selected. After cloning by limiting dilution, hybridomas showed stable murine monoclonal anti-GHS-R production. The monoclonal antibodies subclass was determined as described in the Material and Methods section, most of the Mabs obtained were IgM and we selected the IgM hybridoma: 1D8B2. We used immunoprecipitation to characterize the ability of the monoclonal antibody to recognize the GHS-R expression. Protein extracts of 22RV1 cells, a human prostate cell line kown to express the GHS-R [29], were immunoprecipitated with the selected Mab and with anti GHSR purified commercial polyclonal antibody as control. The western blot analysis were performed with the polyclonal antibody when the monoclonal was used for immunoprecipitation and the other way around; both immunoprecipitation analysis clearly showed a 48 kD band corresponding to the predicted size of GHS-R (Fig. 1A). To characterize this monoclonal antibody we analyzed its ability to detect the specific epitope on the surface of 22RV1 cells by FACS analysis. This analysis demonstrated that the anti-GHS-R Mab recognized the GHS receptor on surface of 22RV1 cells (Fig. 1B). The specificity of our monoclonal antibody was further demonstrated by silencing RNA experiments in which 22RV1 were transfected with GHS-R siRNA. Immunofluorescence staining for GHS-R show significant reduction of signal in transfected cells (Fig. 1C). Moreover, quantitative PCR analysis show a significant reduction in GHSR expression in transfected 22VR1 cells vs non transfected cells (UT, Fig. 1D). In order to test more broadly antibody specificity and reveal eventual cross reactions, a western blot analysis of a selected brain region (hypothalamus) and a non-brain GHS-R bearing tissue (heart) was carried out. Fig. 2A shows a single band of the predicted molecular weight at 48 kDa.

**Figure 3:**
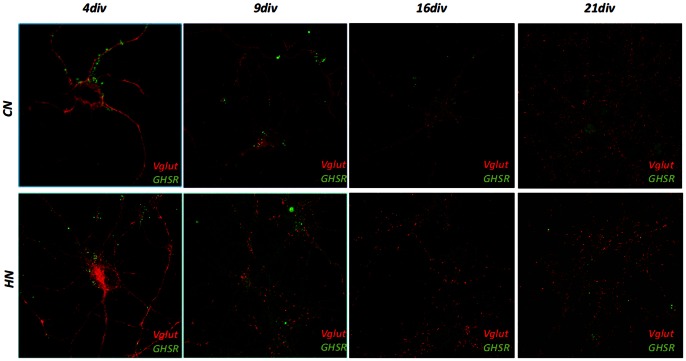
Immunofluorescence staining on primary neuronal cultures. Immunofluorescence staining with GHS-R of either cortical (CN) or hippocampal (HN) neurons at different stages of development. Both neuronal cultures show correct maturation in vitro, as assayed by positive staining of vesicular marker for excitatory neurotransmitter transporter Vglut which shows punctuated expression along neuritis at mature stages of development.

**Figure 4:**
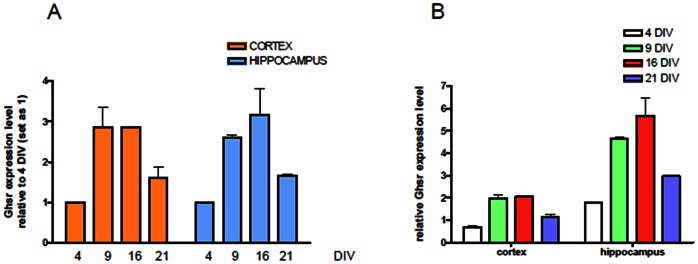
Comparison of GHS-R levels in cortical and hippocampal neurons. A. GHS-R mRNA expression levels in cortical (red) and hippocampal (blue) primary neurons normalized on expression levels at 4 div. GHS-R show a significant increase in expression levels at 9 and 16 div, with a significant reduction at later stages (21 div) in both neuronal populations. B. Relative mRNA expression of GHS-R at different developmental stages of hippocampal (red) and cortical (blue) neuronal cells normalized on GAPDH. GHS-R is significantly more expressed in hippocampal rather than cortical neurons at 9 and 16 div.

At this point, confirmed the specificity of our antibody, we used it to evaluate the expression of GHS-R in the central nervous system. In the rat brain, indeed, detectable levels of GHS-R transcripts have been documented in areas of the hippocampus, the substantial nigra, the ventral tegmental area, the dentate gyrus of the hippocampal formation, and the dorsal and median raphe nuclei [15]. Lysates from hippocampal and cortex neurons, after nine days of culture “in vitro” were immunoprecipitated by 1D8B2 and analyzed by a polyclonal antibody solving a 48 kD band as well as when the immunoprecipitation was performed with a polyclonal antibody was analyzed by a monoclonal (Fig. 2B). Primary neuronal cultures from either rat hippocampus (HN) or cortex (CN) were cultured for different days in vitro (4, 9, 16, 21 div) and immunostained with 1D8B2 MAb. Both neuronal cultures showed correct maturation in vitro, given staining with vesicular marker for excitatory neurotransmitter transporter Vglut show punctuated expression along neuritis branches at mature stages of development. Interestingly, staining for GHS-R showed in both neuronal populations a transient positivity, with a stronger signal at earlier stages of development (up to 9 days) and a significant reduction at later stages (Fig.3). In order to evaluate at which stage of neuronal development there was a greater expression of GHS-R, qRT-PCR experiments were performed on the mRNA from primary rat hippocampal and cortical neurons; the expression of specific mRNA, assayed at 4, 9, 16 and 21 days *“*in vitro” and normalized on the expression at 4 div, showed increasing levels of expression until the reaching of mature stage (16 div) and subsequently a significant decrease at later stages in culture (21 div both in the cortex and in the hippocampus) (Fig. 4A). When expression was normalized with respect to the internal control (GAPDH), in order to compare levels of GHS-R in cortical and hippocampal neurons, a significant higher expression of GHSR in hippocampal rather than cortical neurons was observed, thus suggesting a clear temporal and region-specific expression of the receptor in primary neuronal cultures (Fig. 4B).

## Discussion

In the last few years, the involvement of ghrelin and therefore of its receptors in several physiological and pathological processes has been shown. In light of this, we thought it could be very useful to produce monoclonal antibodies specific for GHS-R. Commercial antibodies specific for GHS-R are all polyclonal antibodies with different indications for the uses, depending on the product, and often limited to just one application such as immunohistochemistry or western blot. The monoclonal antibody produced by us is specific for human and rat GHS-R and our investigations confirm that Mab 1D8B2 is specific for the GHS-R and can be used in immunostaining procedures, such as immunofluorescence, immunoprecipitation, western blot and FACS analysis. Moreover FASTA analysis demonstrated that the chosen peptide sequence, which have been used to immunize mice, did not share any homology with other human/rat protein. For the characterization of this antibody we used a line of human prostate cancer, described as positive for GHSR, in immunoprecipitation and flow cytometry experiments (Fig. 1A). These analysis showed that the monoclonal antibody we obtained was specific for GHS-R. Moreover the specificity of our monoclonal antibody was demonstrated by silencing RNA experiments in which GHS-R expressing prostate cell line was transfected with GHS-R silencing RNA. Immunofluorescent staining for GHS-R show significant reduction of signal transfected cells. We then focused our attention on the brain tissue, more specifically on two selected and distinct brain areas namely hippocampus and cortex. By immunoprecipitation, we identified the presence of GHS-R in both tissues. The immunostaining of both cortical and hippocampal neurons with 1D8B2 Mab enables us to observe increasing levels of GHS-R expression in developing neurons, which significantly reduces in mature neurons at longer times in cultures, representative of a developed neuronal network enabling aging studies [30]. This transient behavior correlated with an increase in mRNA expression during development and a subsequent decrease in older cultured neurons. Interestingly, a sustained mRNA expression at 16 div does not correlate with a sustained marker positivity by IF, thus suggesting a potential modulation of receptor insertion in the membrane (i.e. internalization) which will be further elucidated in future studies.

When comparing cortical and hippocampal levels of GHS-R mRNA normalized to GAPDH, it was clearly noted a significantly higher expression in the hippocampal with respect to cortical neurons. This observation is of extreme importance given not only it indicates that GHS-R expression in primary neurons is modulated in a time-dependent manner, but that there might also be a selective regional distribution which might yield to functional modulatory effects of ghrelin in the two brain areas.

Further studies are needed in order to characterize the effects induced by ghrelin in selected neuronal populations at different developmental stages. For example, it is our intention to use this antibody to investigate the role of ghrelin in aging, as it seems that some phenomena typical of aging, such as decreased memory [31], [32] and the role of this receptor in some neurodegenerative diseases such as Alzheimer's disease.
